# Periodicities in an active region correlated with Type III radio bursts observed by Parker Solar Probe

**DOI:** 10.1051/0004-6361/202039510

**Published:** 2021-06-02

**Authors:** Cynthia Cattell, Lindsay Glesener, Benjamin Leiran, John Dombeck, Keith Goetz, Juan Carlos Martínez Oliveros, Samuel T. Badman, Marc Pulupa, Stuart D. Bale

**Affiliations:** 1 School of Physics and Astronomy, University of Minnesota, 116 Church St. SE Minneapolis, MN 55455, USA; 2 Space Sciences Laboratory, University of California, Berkeley, Berkeley, CA 94709, USA; 3 Department of Physics, University of California, Berkeley, Berkeley, CA 94709, USA

**Keywords:** Sun: radio radiation, Sun: corona, Sun: X-rays, gamma rays, Sun: oscillations, magnetic reconnection, radiation mechanisms: non-thermal

## Abstract

**Context.:**

Periodicities have frequently been reported across many wavelengths in the solar corona. Correlated periods of ~5 min, comparable to solar *p*-modes, are suggestive of coupling between the photosphere and the corona.

**Aims.:**

Our study investigates whether there are correlations in the periodic behavior of Type III radio bursts which are indicative of nonthermal electron acceleration processes, and coronal extreme ultraviolet (EUV) emission used to assess heating and cooling in an active region when there are no large flares.

**Methods.:**

We used coordinated observations of Type III radio bursts from the FIELDS instrument on Parker Solar Probe (PSP), of EUV emissions by the Solar Dynamics Observatory (SDO) Atmospheric Imaging Assembly (AIA) and white light observations by SDO Helioseismic and Magnetic Image (HMI), and of solar flare X-rays by Nuclear Spectroscopic Telescope Array (NuSTAR) on April 12, 2019. Several methods for assessing periodicities are utilized and compared to validate periods obtained.

**Results.:**

Periodicities of ~5 min in the EUV in several areas of an active region are well correlated with the repetition rate of the Type III radio bursts observed on both PSP and Wind. Detrended 211 and 171 Å light curves show periodic profiles in multiple locations, with 171 Å peaks sometimes lagging those seen in 211 Å. This is suggestive of impulsive events that result in heating and then cooling in the lower corona. NuSTAR X-rays provide evidence for at least one microflare during the interval of Type III bursts, but there is not a one-to-one correspondence between the X-rays and the Type III bursts. Our study provides evidence for periodic acceleration of nonthermal electrons (required to generate Type III radio bursts) when there were no observable flares either in the X-ray data or the EUV. The acceleration process, therefore, must be associated with small impulsive events, perhaps nanoflares.

## 1. Introduction

Quasi-periodic variations with periods ranging from seconds to tens of minutes have long been reported for many phenomena in the active and quiescent solar corona, starting from the first detection of correlated periodicities in solar flare X-rays and microwaves (Parks & Winckler 1969). Examples include rapid variations in Type III radio bursts (Mangeney & Pick 1989; Ramesh et al. 2005). In the extreme ultraviolet (EUV), the extensive observations of these periodicities, which are often interpreted as the signature of magnetohydrodynamic (MHD) waves, have led to the development of coronal seismology, to assess properties of the corona (Nakariakov & Verwichte 2005; Kupriyanova et al. 2020; Roberts et al. 1984; Roberts 2000; De Moortel & Nakariakov 2012). Long-lived 3 to 5 min pulsations are also observed in sunspots (Sych et al. 2012, 2020; Battams et al. 2019). Quasi-periodic variations in X-rays and gamma-rays from solar and stellar flares are also observed (Van Doorsselaere et al. 2016; Kupriyanova et al. 2020; Inglis et al. 2015; Dennis et al. 2017; Hayes et al. 2020).

Several studies have described correlated periodicities in various combinations of Type III radio bursts, hard X-rays, EUV emissions, microwaves, and sunspots at periods of minutes. Type III bursts are of particular interest because they provide information on acceleration of nonthermal electron beams. Innes et al. (2011) reported correlations between ~3 min periodicities in Type III radio bursts and coronal jets observed in 211 Å, which were possibly related to 3-min. oscillations in sunspot brightness. Most other studies have described periodicities in association with large flares. Oscillations in Type III radio waves, hard X-rays, and jets at ~4 min periods were reported by Li et al. (2015). Kumar et al. (2016) found ~3 min. pulsations in hard X-rays, microwave emission, Type III bursts and a nearby sunspot.

Foullon et al. (2010) found periods of ~10 min in Type IIIs and X-rays, with longer ~18 min periods in the few gigahertz radio emissions, which are interpreted to be due to nonthermal gyroresonance. Shorter periods (~100 s) were reported by Kumar et al. (2017) in correlations between coronal fast mode waves, Type III and IV radio bursts, microwaves, and thermal X-rays. Kupriyanova et al. (2016) described ~40 s variations in hard X-rays, microwaves and Type III waves, consistent with a modulation of nonthermal electron acceleration, but not thermal processes.

Explanations for the periodicities include MHD waves (Alfvén waves and fast or slow mode magnetosonic waves), modulation of reconnection at flare sites via intrinsic processes, current sheet structure (De Moortel & Nakariakov 2012; McLaughlin et al. 2018; Aschwanden 2006), or coupling of solar p-modes to coronal waves (Zhao et al. 2016). For events that include nonthermal X-rays and/or Type III radio bursts, periodicities are most often attributed to modulated reconnection. The periodic behavior which is observed in theoretical and modeling studies of reconnection may be intrinsic and due to loading and loading, or due to modulation by MHD waves, or due to other processes, such as those described by Murray et al. (2009), Heggland et al. (2009), McLaughlin et al. (2012), Thurgood et al. (2017). An alternate approach attributes quasi-periodic behavior to stochastic processes (Veronig et al. 2000; Aschwanden et al. 2016; Eastwood et al. 2009).

In this report, we describe observations of repetitive Type III bursts observed by Parker Solar Probe (PSP) on April 12, 2019, and their correlation with periodic rapid heating and cooling in the 211 and 171 Å bandpass filters (~2 MK and ~0.6 MK peak temperature responses, respectively) on the Solar Dynamics Observatory (SDO)/Atmospheric Imaging Assembly (AIA). Section 2 describes the data sets and analysis techniques; Sect. 3 shows the observations; and Sect. 4 discusses the results, comparisons to other studies, and an interpretation in terms of possible physical models.

## 2. Data sets

We focus on an interval on April 12, 2019, when simultaneous data were obtained by NuSTAR, PSP, Wind, and SDO. Radio data were obtained with the Radio Frequency Spectrometer (RFS) (Pulupa et al. 2017), which is part of the PSP FIELDS suite (Bale et al. 2016). This interval is at the end of the second encounter, so the sample rate was low, ~1 sample per 55 s. We also examined higher rate data at 1 sample per 16 s for intervals with similar periodic bursts. We also utilized radio data (1 sample per 7 s) from RAD1 (20–1040 kHz) and RAD2 (1.075–13.825 MHz), which are part of the Wind/Waves (Bougeret et al. 1995)

Extreme ultraviolet data from SDO Atmospheric Imaging Assembly (AIA; Lemen et al. 2012) and magnetic field information from the Helioseismic and Magnetic Imager (HMI; Schou et al. 2012) were utilized to examine periodicities and solar structures. AIA data were obtained in seven wavelengths at a 12 s cadence over the full sun. Analysis of AIA data is focused on five areas within active region 12 738 (NOAA designation).

During the interval of interest, based on field line tracing using a combination of the Potential Field Source Surface model and the Parker spiral (see Badman et al. 2020 for a description of the method), PSP and Wind both map closer to the smaller active region near 60 degrees longitude, which was not observable by SDO at this time. Harra et al. (2020) study this smaller active region (AR 12 737) and conclude from the dynamics that it may be contributing to the population of Type III bursts observed, although they study an earlier interval from March 31–April 6, during which time AR 12 738 was behind the limb as viewed from Earth. PFSS mapping also suggests that there was no direct magnetic connection to either active region and thus no in situ observations of electron beams are expected (or indeed observed) at PSP or Wind. One study (Krupar et al. 2020) used radio triangulation with STEREO and Wind to show that in at least one case study of a large Type III burst, the electron beam appears to be consistent with a Parker spiral emerging from AR 12 378. Pulupa et al. (2020), who examined the full encounter, also associated the bursts with AR 12 738, citing the consistency of the orientation of its bipole on the solar disk with the polarization of radio emission measured in situ (although this is also true of AR 12 737 since it is in the same solar hemisphere in the same solar rotation). The observations of Krupar et al. (2020) and Pulupa et al. (2020) suggest that our comparison of the Type III bursts to periodicities in AR 12 738 is appropriate.

**Fig. 1.**
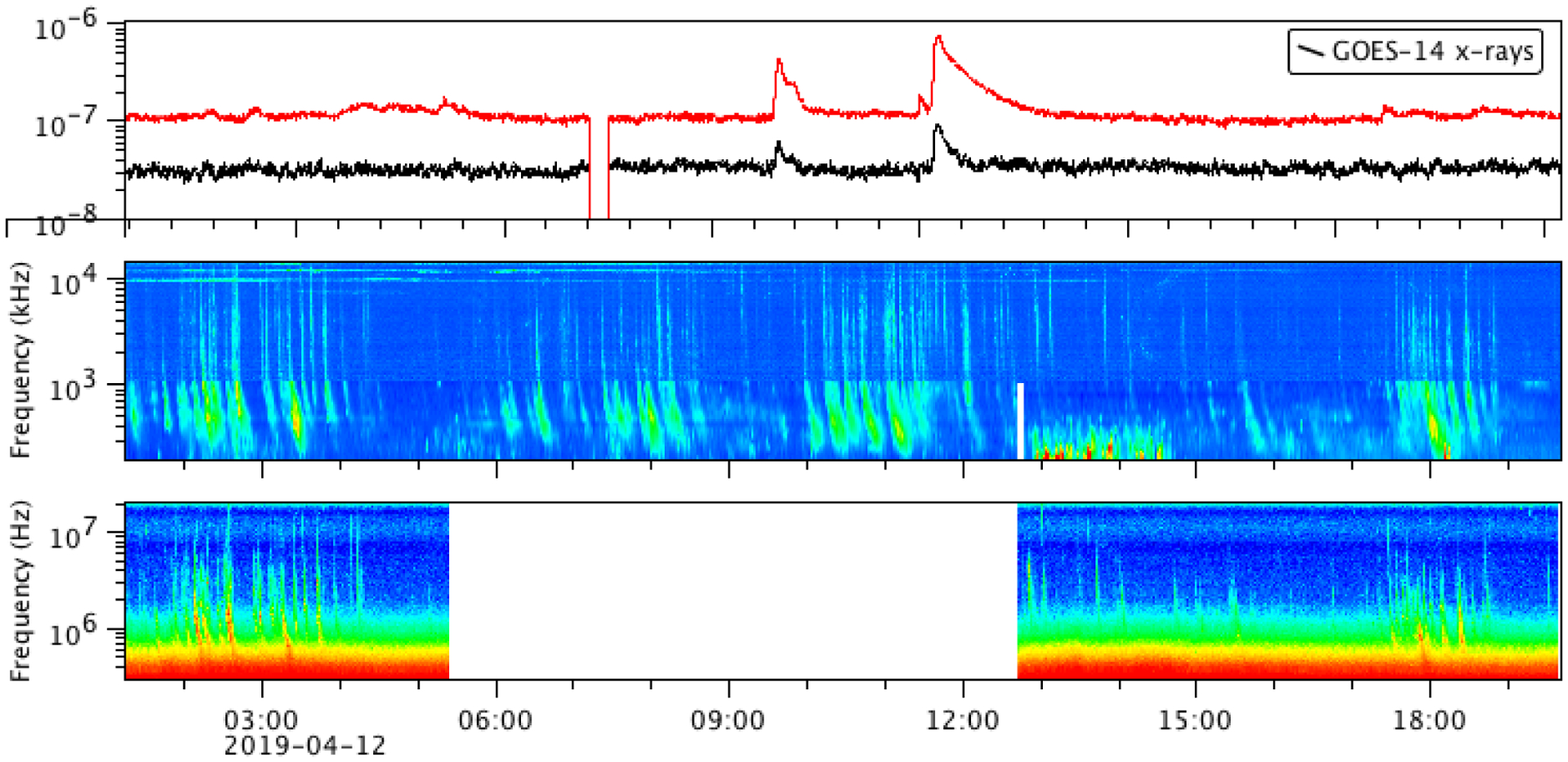
Overview of (*top*) X-rays observed by GOES-14, and Type III radio bursts (300 kHz to 20 MHz) observed by (*middle*) Wind and (*bottom*) Parker Solar Probe for April 12, 2019 0100 to 2000 UT. For the GOES-14 X-rays, the red line is 0.5–4 Å (3–24 keV) and the black line is 1–8 Å (1–12 keV). Times are those at which the emission was recorded on the respective spacecraft; the PSP times have not been shifted to 1 AU.

**Fig. 2.**
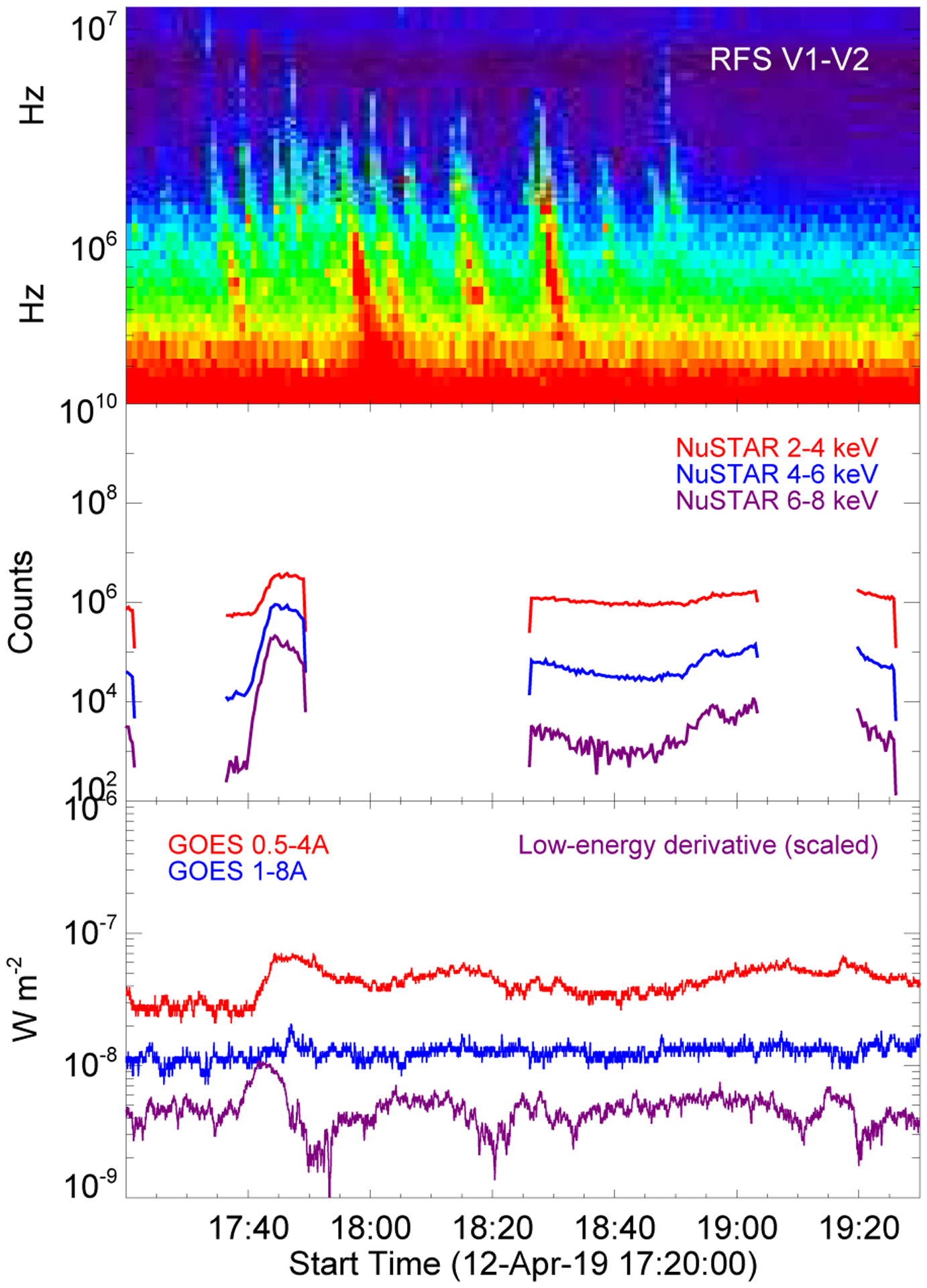
Overview of the interval of interest from 17:20 to 19:30 UT. *Top panel*: PSP/FIELDS radio data (V12), with times propagated to 1 AU. *Middle panel*: NuSTAR X-ray data, and *bottom panel*: GOES X-ray data.

**Fig. 3.**
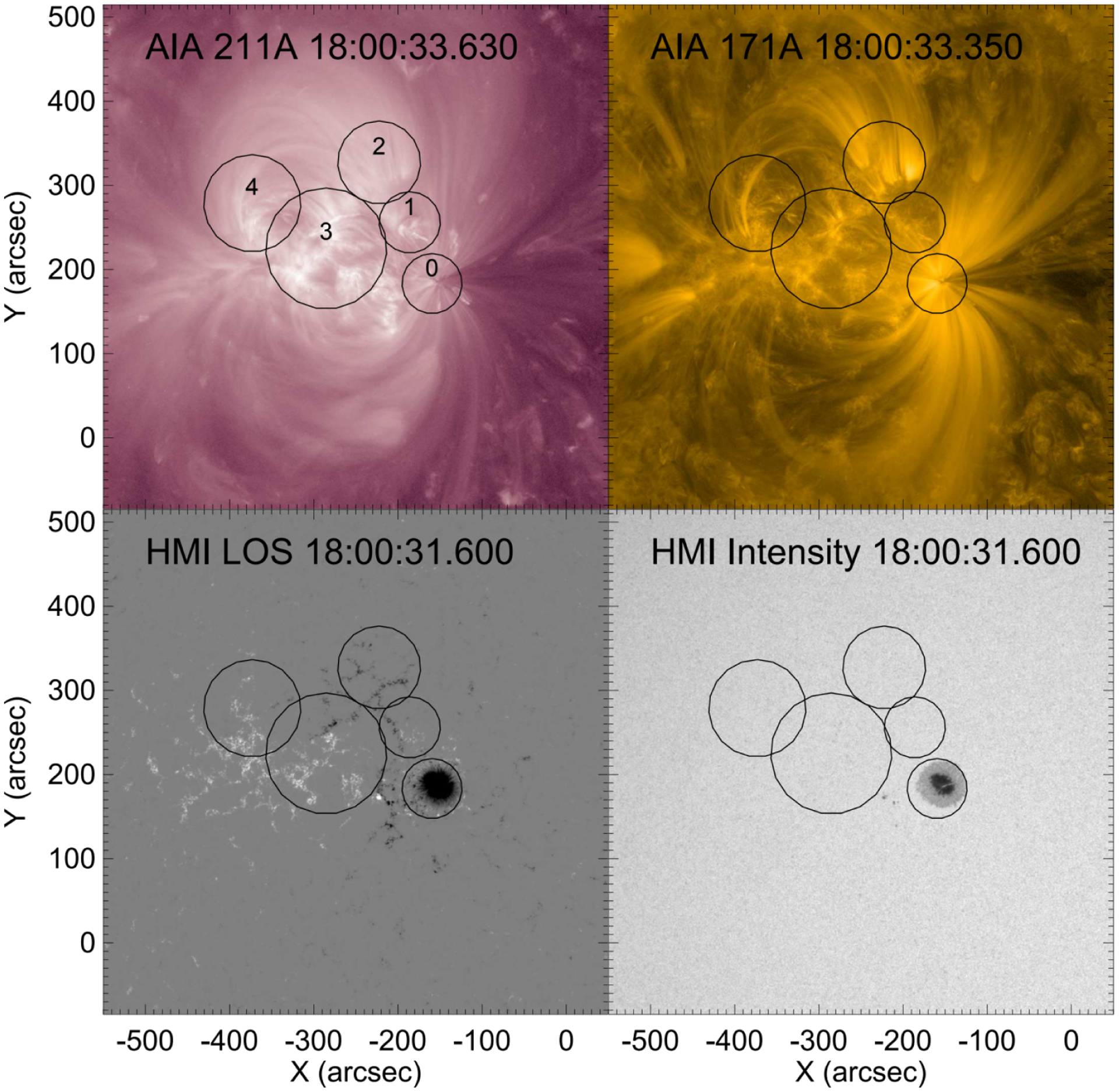
Regions analyzed overlaid on SDO images. *Top row*: two AIA filters that have sensitivity to quiescent coronal temperatures, and *bottom row*: HMI line-of-sight magnetogram and intensity map. Images shown are at a time near the middle of the analyzed interval. At the latitude of this active region, solar rotation causes sources to drift westward at a rate of about 10 arcsec per hour.

On April 12 and April 13, 2019, at the end of the second PSP periapsis pass, NuSTAR observed the sun, measuring hard X-rays at energies from 2.5 to 10 keV for six subintervals when PSP obtained radio data. Several GOES A7 to A9 class flares were seen in each of the two intervals on April 12, 2019 for which there are PSP radio data, and two B class flares on April 13, 2019 with PSP radio data. See Figs. 1–3 for summaries of the observations.

## 3. Observations

An overview of the event is shown in Fig. 1, including the X-ray data from GOES-14 (panel a), as well as the Type III radio bursts seen on Wind (panel b) and PSP (panel c). During the interval from 540 UT to 1230 UT, all PSP instruments were turned off to enable high rate science data downlink. The quasi-periodic repetition of the Type III radio bursts seen by both PSP and Wind is clear, with periods of ~4 to 5 min. We note that similar bursts were observed intermittently for ~10 days from either or both of PSP and Wind. The interval displays some characteristics of a Type III radio storm (Fainberg & Stone 1970; Bougeret et al. 1984; Morioka et al. 2007). The individual bursts are lower power and have a more limited frequency range than flare-associated Type IIIs. The repetition rate is longer than the range found for storms (~10 s to 1 min); however, this may be due to the low level of solar activity. Pulupa et al. (2020) characterize the properties of the Type III bursts for two intervals, one before our observations (April 3, 2019 08 UT to April 4, 2019 08 UT), and one after (April 17,2019 17:00 UT to April 18,2019 05:00 UT), and conclude the Type IIIs were associated with Type III storms.

To examine possible correlations between the low corona (as probed by AIA) and the Type III radio bursts, we focused on the interval between 1715 and 18:45 UT, which is the first time period when NuSTAR data were obtained. Figure 2 plots the PSP radio data, the NuSTAR X-rays, and the GOES X-rays together, with PSP times shifted to 1 AU. Although the flares do occur during times of intense radio activity and the X-ray bursts may be related to the radio bursts, there is not a one-to-one correspondence between the flare X-ray emissions (c and d) and the Type III bursts.

**Fig. 4.**
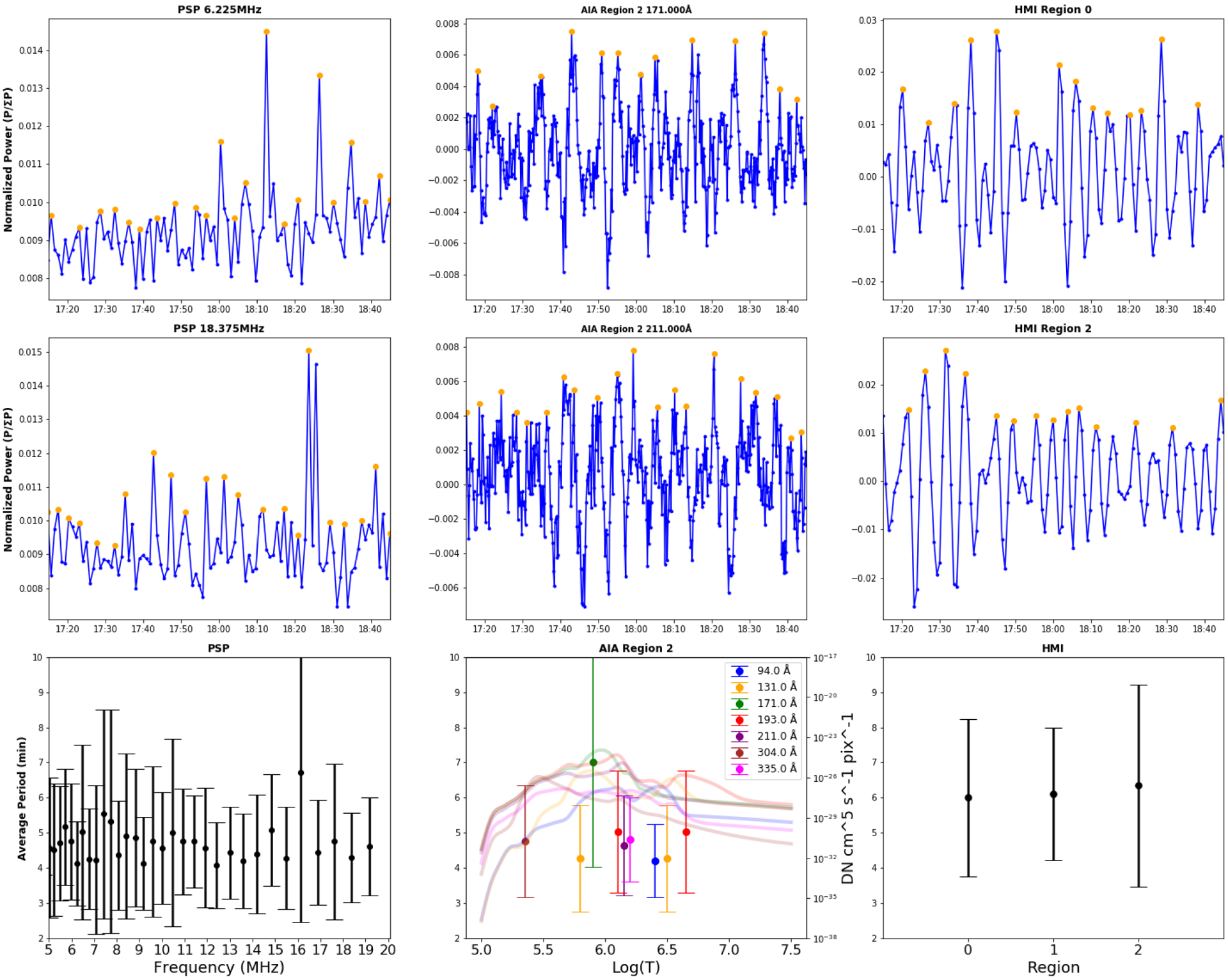
Periodicities in PSP/FIELDS HFR radio power, AIA emission in Region 2 (as defined in Fig. 3), and HMI intensity. (*a*) 6.225 MHz and (*b*) 18.375 MHz interpolated normalized power with black dots indicating the identified peaks; (*c*) periods identified for all frequencies >5 MHz; (*d*) AIA 171 Å and (*e*) 211 Å detrended normalized intensity with black dots indicating the identified peaks; and (*f*) periods identified for all AIA passbands in Region 2, versus wavelength with temperature response curves overplotted; (g) and (*h*) HMI emission in two regions; and (*i*) periods identified in HMI data. *Panels d and e*: AIA lightcurves have been detrended. The units are residual data numbers (DNs) after a 10-minute average curve was subtracted. *Panel f*: temperatures of the data points are those listed in Table 1 of Lemen et al. (2012) for each passband (these are approximately the peaks of the temperature responses), but the colored lines show the temperature responses themselves, in arbitrary units, to give a better representation of possible temperatures for each data point. When more than one temperature is listed in Table 1 of Lemen et al. (2012), we have included a data point for both, since we cannot distinguish them.

SDO/AIA measures EUV emission from the Sun in passbands defined by ten filters, six of which are sensitive to coronal temperatures (Lemen et al. 2012). Images are full-Sun at 1.2 arcsec resolution and a 12 s cadence for each filter. Several diverse regions of active region 12 738 were selected for individual analysis, including the region at the major sunspot, regions where small transients were visible by eye, and some quiet regions. Regions are shown in Fig. 3. Within each of these regions, AIA emission in individual filters was totaled over the region and plotted as a function of time. These included all of the coronal filters as well as the 304A filter, which is sensitive to the He II ions typically found in the chromosphere and transition region. Solar rotation was not removed, so the solar emission drifts across each region at a slow rate (~10 arcsec per hour). Some regions exhibited a periodic behavior to their time profiles in addition to macroscopic, transient events. The time profiles were detrended so that this periodic behavior was more apparent, as shown in Fig. 4, to be discussed in detail below, panels d and e. Detrending was performed by subtracting a smoothed curve (with a running average over 10 min) from each time profile.

Figure 3 shows the active region with the five subregions indicated. The top panels are images of 211 A and 171 A from AIA, and the bottom panels are the line-of-sight (LOS) magnetogram and intensity map from HMI. For the EUV images, our study utilizes bandpass filter images, and as such examines only periodicity in the EUV bandpass brightnesses, and not in other properties such as line Doppler velocity or Doppler width.

The EUV and radio data were examined for periodicities utilizing a method that identified peaks and valleys above a threshold value in the normalized power (PSP and Wind) or normalized detrended light curve (SDO/AIA and HMI). The detrending time periods and intervals for analysis of AIA data were selected to avoid introducing artificial periods (Auchère et al. 2016; Dominique et al. 2018). Periodicities for the radio data were determined both using the measured values and using data interpolated to the cadence of the AIA data. Figure 4 shows an example of the “peak” approach for the radio, EUV and HMI data sets. Panels a and b show the normalized power above average for the two frequency bands (6.2 and 18.4 MHz) from the PSP radio data versus time. Panel c plots the resulting average periods versus frequency for the HFR (>5 MHz) band, which was determined using a threshold of one for the normalized power. Panels d and e plot the detrended normalized intensity for two AIA bands, 171 and 211 Å in Region 2; and panel f plots the average period versus temperature (based on the peak of the temperature responses of each bandpass for the six coronal lines and the one photospheric line) for Region 2, where the periodicities were most prominent. The AIA temperature responses are overplotted; colors identify the same lines for both periodicity and temperature response. It should be noted that all of the AIA bandpass filters have broad temperature responses and some have bimodal responses. We cannot use these data to measure a strict temperature without differential emission measure analysis, but the peak temperature response gives a rough estimate of which temperature range we are likely to be observing. For the filters that have a doubly peaked response, we plotted the temperatures of both peaks for reference. Although some studies of quasi-periodicities in EUV lines have identified a temperature dependence, the periods we observe are independent of temperature to within the error bars. Panels g and h plot the time series of the HMI intensity for two regions within the EUV subregion 0, and the periods determined for all three HMI regions are plotted in panel i. It is clear that the periodicity in the radio waves (~4–5 min) is comparable to those in the AIA and HMI data. We note that periodicities in the radio, EUV and HMI data were also determined using fast Fourier transforms (FFTs), with similar results.

**Fig. 5.**
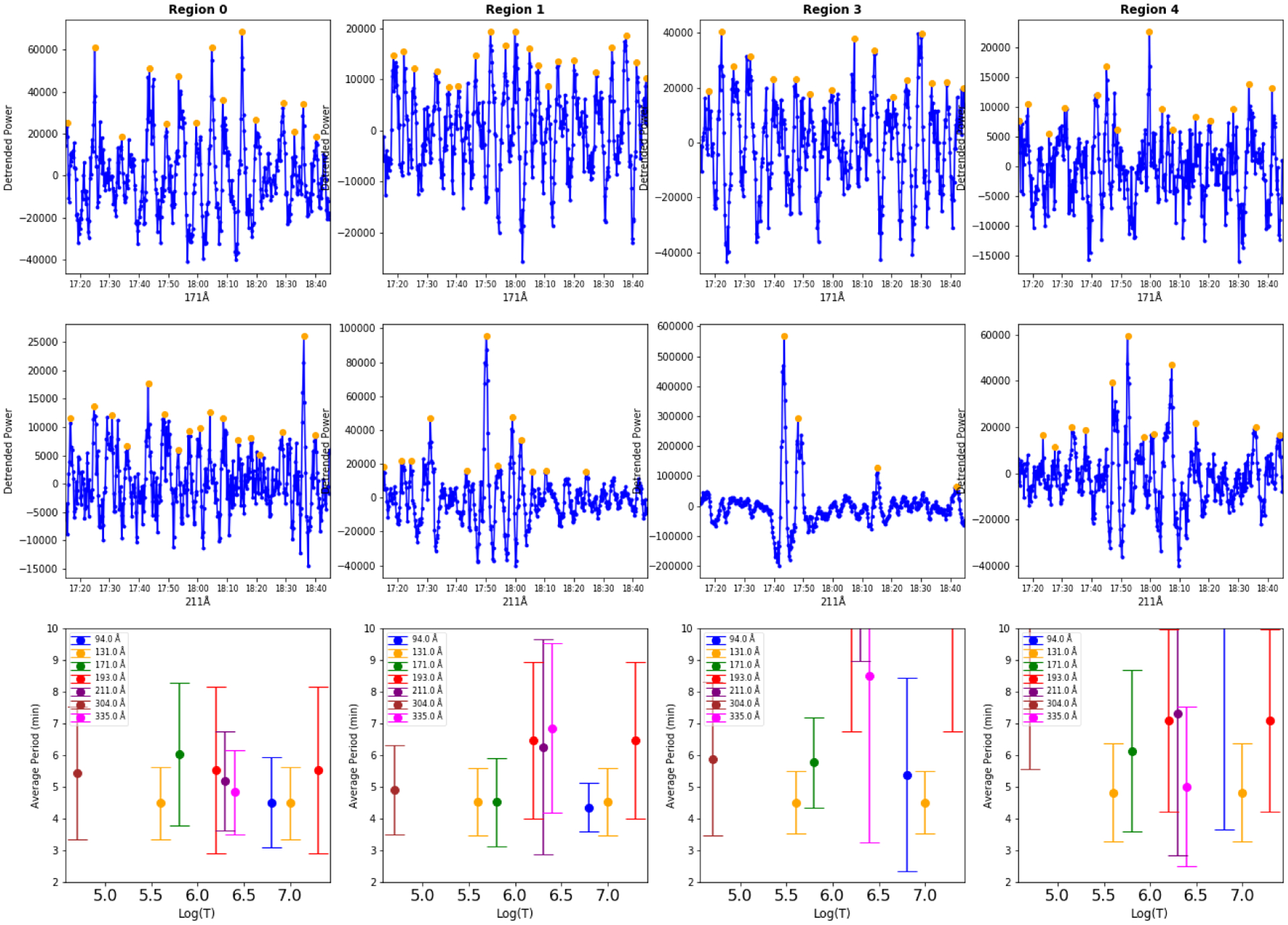
Periodicities in the AIA emission in Regions 0, 1, 3 and 4 (as defined in Fig. 3) The format is the same as in Fig. 4 panels d–f.

The behavior of the EUV emission in the other four regions was also examined (see Fig. 5). The amplitudes of the variations in the light curves are smaller, which results in larger error bars in the determination of periods. In most wavelength bands and regions, the periodicities are similar to those seen in Region 2. In Regions 1, 3 and 4, periods in the 193, 211, and 335 Å bands are slightly longer; however, given the large error bars, it is not possible to reach any definitive conclusions about differences in periodic behavior between the regions.

**Fig. 6.**
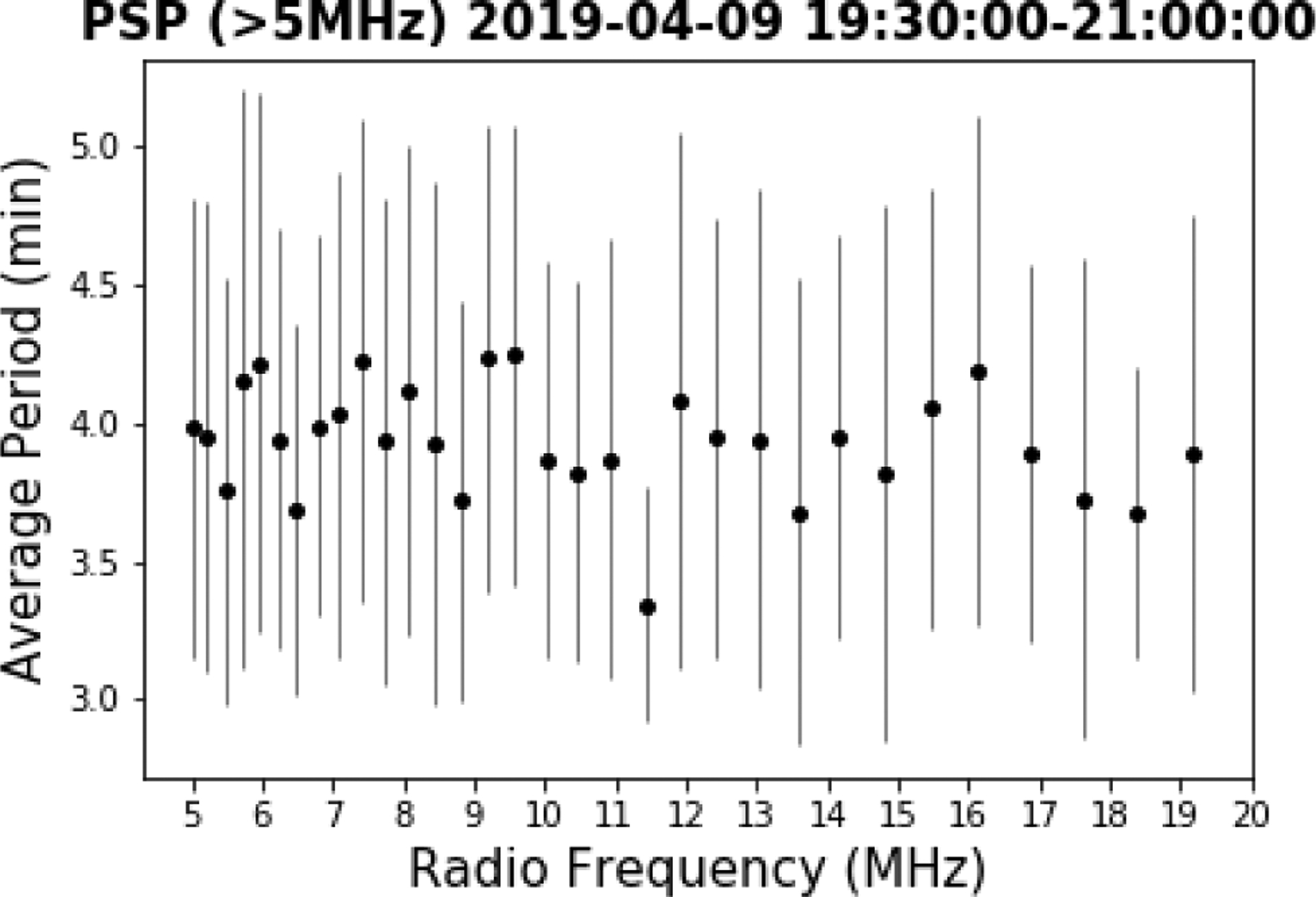
Periodicities seen in PSP radio for an earlier interval with higher data rate.

We have also examined periodicities in the PSP radio data for intervals with similar repetitive Type III bursts earlier in this pass when higher rate data were obtained. Figure 6 (same format as Fig. 4) shows that the periodicities observed in the Type IIIs are very stable, and they are observed for many days. Repetitive Type III radio bursts observed by PSP earlier in this encounter are discussed by (Harra et al. 2020).

**Fig. 7.**
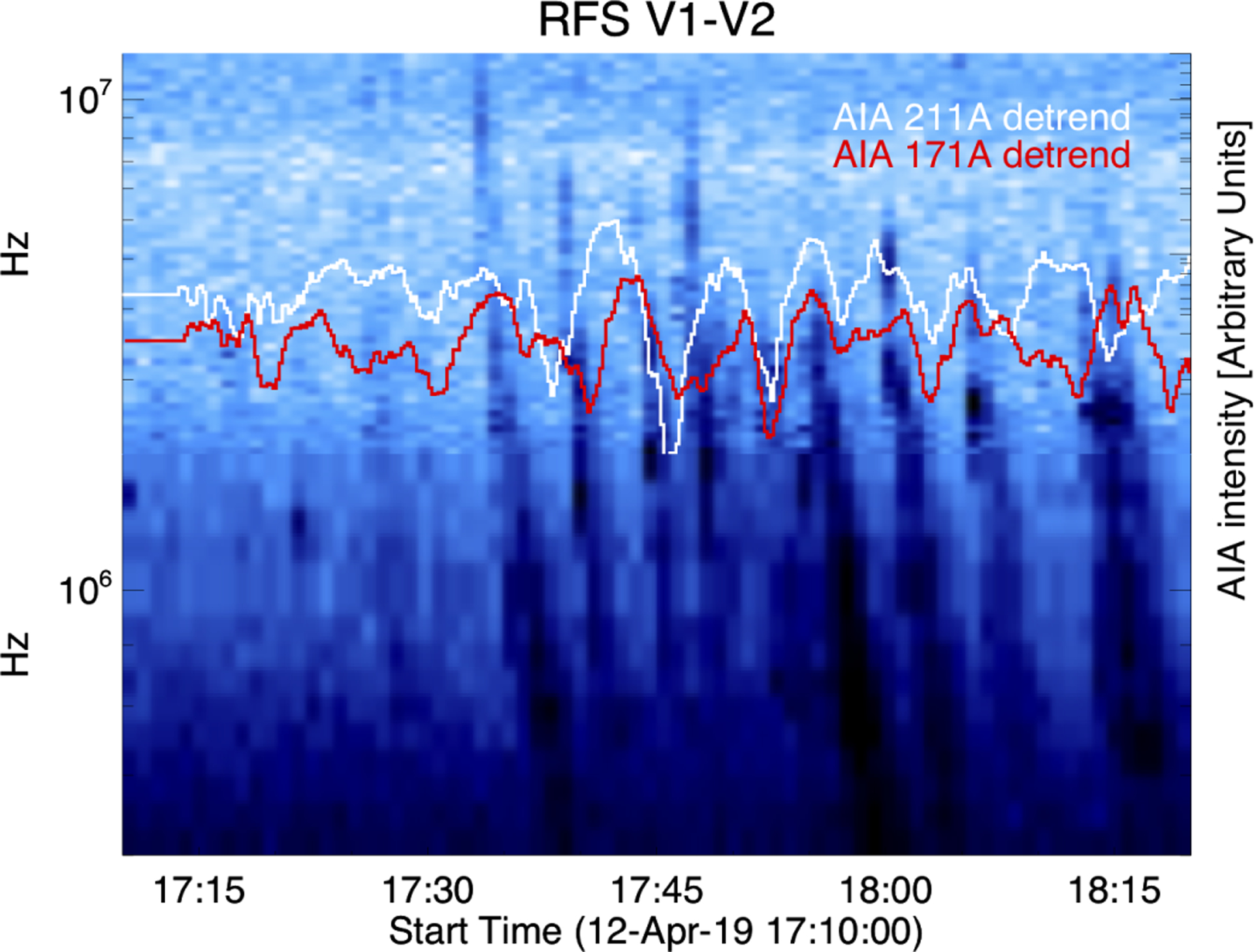
Time series of PSP radio with detrended AIA 211 Å (white) and 171 Å (red) overplotted for Region 2. PSP times were propagated to 1 AU.

The correlation between the times series of the PSP radio data and the SDO/EUV data is shown in Fig. 7, in which the 171 Å (in red) and 211 Å (in white) detrended light curves are plotted on top of the PSP radio power. The radio data were time-shifted to account for propagation from the solar radial position of PSP to 1 AU. As is evident in the figure and as would be expected from the physics of Type III bursts, the delay times between the EUV lines and the radio bursts vary with frequency. Although the radio burst onset often leads the 211 Å which often leads the 171 Å as can be seen in the figure for the first three radio bursts after 1730, for some bursts the relationship is different. For the next set of radio bursts, the radio and EUV peaks are closer together in time, and the time ordering varies. The association qualitatively observable in the figure is confirmed using cross-correlation analysis. The correlation coefficient between the two EUV lines is typically larger than that obtained between the radio data and the EUV lines, due to the lower sample rate for the radio data. From ~1740 to ~1800, the 211 Å line leads the 171 Å line with the lead time decreasing from ~100 to 0 s (correlation coefficient of ~0.7 to ~0.8); the 3.5 MHz band leads the 211 Å line by an amount increasing from ~50 to 250 s (correlation coefficient of ~0.4 to ~0.6). From ~1800 to the end of Fig. 7 (1820), the 211 Å line lags the 171 Å line, although after ~1810 the correlation coefficient for the 211 Å leading the 171 Å is comparable. We note that the interval where the time ordering of the two EUV lines changes is when the 171 Å light curve has a clear double peak, while the corresponding signal in 211 Å has one large peak preceded by a smaller peak. From ~1745 to 1820, the lower frequency radio bands (several meghertz) lead the 211 Å line. We note that the power at higher frequencies is lower after ~1800, so the cross-correlation between the higher frequency radio bands and the EUV is weaker than for the lower frequencies. The average cross-correlation over the entire time period between the two EUV lines is ~0.4, with 171 Å lagging the 211 Å by 72 s. The average correlation between the 211 Å line and the 171 Å line as well as the strong correlation observed between ~1740 to ~1805 are suggestive of rapid heating, followed by rapid cooling. A possible explanation for the varying lags between these data sets could be that heating events are occurring on flux tubes of different initial temperatures and densities. For a coronal density on the order of 10^9^ cm^−3^, the time necessary to ionize to the Fe XIV state (the main ion to which AIA 211 Å is sensitive) is on the order of tens to hundreds of seconds between 1 and 2 million degrees, while for hotter temperatures and higher densities this time delay is negligible. (These calculations were performed using the CHIANTI database, version 9.0.1.) The fact that the radio bursts usually lead the enhancements in the 211 Å line is consistent with a possible interpretation that the heating is due to electrons accelerated that are downward in the small-scale reconnection that accelerates electrons upward to generate the radio waves.

**Fig. 8.**
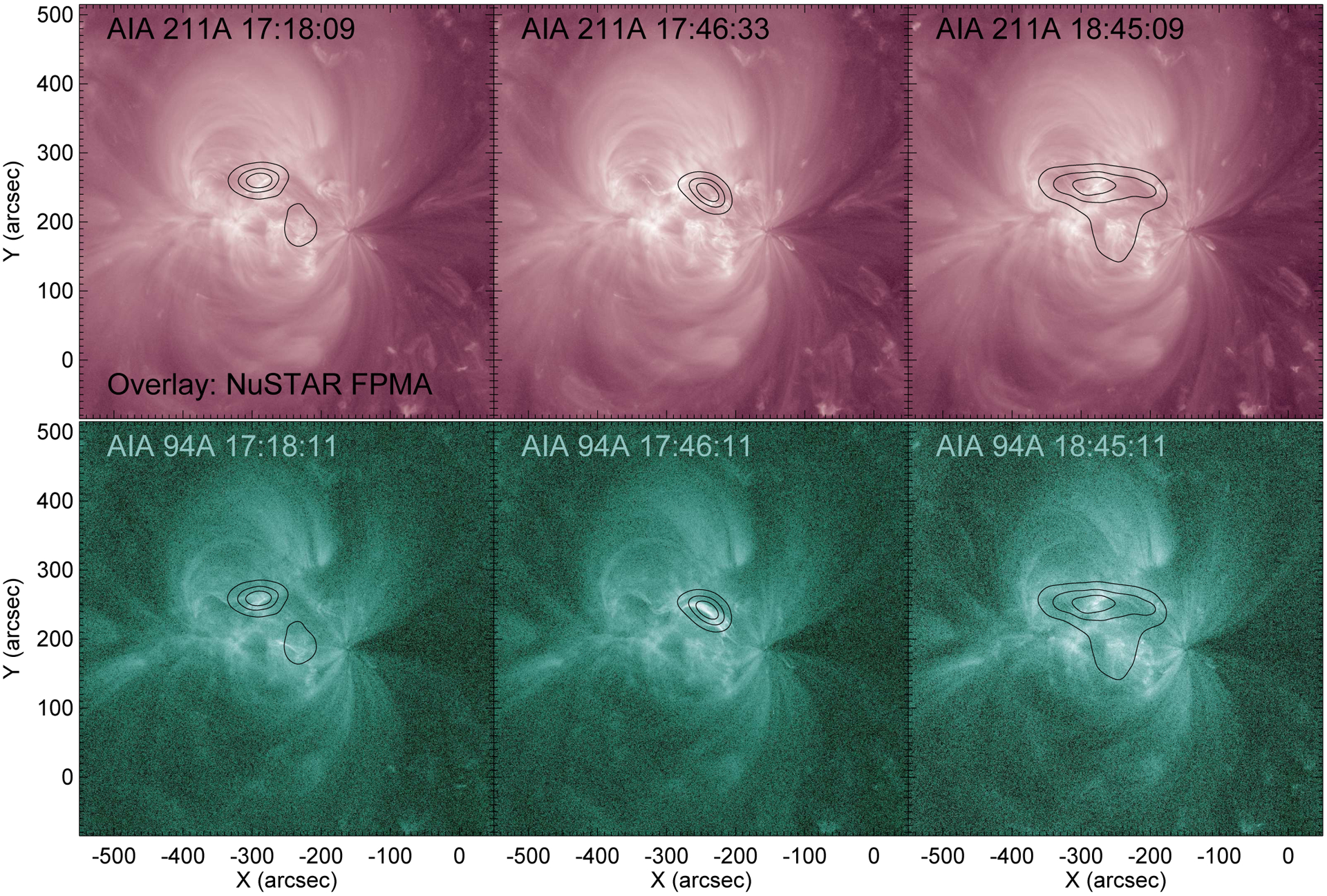
NuSTAR emission from 2 to 6 keV overlaid on AIA images in the (*top*) 211 Å and (*bottom*) 94 Å bandpass filters. The times of integration for the NuSTAR images are 17:15 to 17:21 UT, 17:44 to 17:49 UT, and 18:40 to 1850 UT in the *left, center, and right columns* respectively. NuSTAR images were deconvolved for ten iterations. As discussed in the text, there is uncertainty in the coalignment of NuSTAR and AIA emission.

We examined the NuSTAR soft and hard X-ray emission for evidence of pulsations. NuSTAR time profiles are shown the middle panel of Fig. 2 and NuSTAR images are shown in Fig. 8. For the latter, emission from NuSTAR’s focal plane module (FPM) A was integrated over three time periods, including flaring and nonflaring times. Images from FPMB (not shown) look similar to those from FPMA. The point spread function of the instrument was deconvolved from the images using the IDL procedure max_likelihood, with ten iterations of deconvolution. Due to large pointing uncertainties when NuSTAR is Sun-pointed (see Grefenstette et al. 2016), images were coaligned to AIA data so that image centroids matched bright features in the AIA 94 and 335 Å filters. For the time of the microflare (middle column in Fig. 8), this coalignment is good to approximately 5 arcsec since there are bright compact features available to coalign. At other times, the uncertainty is on the order of 30 arcsec, since clear, compact features available are not available for coalignment. Although Fig. 2 seems to show some periodic variations in the X-ray time profiles by eye, it was not possible to isolate periodic behavior similar to that observed in AIA and FIELDS data, for two (related) reasons. First, the presence of small (GOES A class) microflares, for example the one from 17:40 to 17:45 UTC, dominates NuSTAR’s high sensitivity. Second, since the coalignment of NuSTAR with AIA has large uncertainties at nonflaring times, it is not possible to isolate emission from the same regions shown in Fig. 3 with a high degree of confidence. Additionally, NuSTAR’s point spread function wings make it difficult to isolate nonflaring emission when activity is present anywhere in the region. In summary, we do not find positive evidence of pulsations with few-minute periods in NuSTAR data, but they cannot be ruled out either.

## 4. Discussion

Many different mechanisms have been proposed to explain observed periodic and quasi-periodic behavior in the EUV and radio data. Our study provides evidence for periodic acceleration of nonthermal electrons (required to generate Type III radio bursts) when there were no observable flares either in the X-ray data or the EUV. The occurrence of Type III bursts without significant flaring was also observed by Harra et al. (2020) earlier in this PSP encounter. The acceleration process, therefore, must be associated with small impulsive events (perhaps nanoflares; Viall & Klimchuk 2011; Bradshaw et al. 2012; Ishikawa et al. 2017; Hudson 1991; Klimchuk 2015), or with some other mechanism such as kinetic Alfvén waves (McClements & Fletcher 2009). Small acceleration events have also been seen as isolated events (James et al. 2017) with a Type III burst and only very weak hard X-rays. If the mechanism is nanoflares, the electron acceleration may involve processes seen in flares (e.g. those described in Zharkova et al. 2011). Studies of small microflares using NuSTAR have provided evidence for acceleration of nonthermal electrons at energies below 7 keV (Glesener et al. 2020; Duncan et al. 2020), with significant collisional energy deposition that could provide heating of the corona. Indications of accelerated electron distributions also come from indirect, but highly sensitive, measurements of the transition region’s response to those electrons, as studied by Testa et al. (2014, 2020). These studies show evidence of accelerated electrons even in extremely small events that cannot be studied using more direct methods yet. Studies have predicted a range of periodicities for nanoflares (Viall & Klimchuk 2011; Bradshaw et al. 2012; Klimchuk 2015; Knizhnik & Reep 2020) depending on parameters, such as cooling rates.

Many researchers have developed models of reconnection that result in periodicities of minutes. The simulations of Murray et al. (2009) showed that oscillatory reconnection with a period of a few minutes was associated with bounded outflow regions. Similar inherent periodic behavior was described by McLaughlin et al. (2012). Chen & Priest (2006) concluded that reconnection driven by *p*-mode oscillations could result in rapid temperature changes in the outflowing plasma. In simulations of reconnection driven by photospheric waves, Heggland et al. (2009) explicitly computed synthesized emissions in the EUV. They discussed several possible explanations for the complex relationships between brightening in different lines. Their study may provide clues as to the changing time ordering of the 171 Å line, the 211 Å line, and the radio peaks that we observed.

One possibility for obtaining periodicities is that MHD waves in the corona can initiate magnetic field reconfiguration, resulting in small-scale reconnection. It has been shown that propagating coronal waves can destabilize active regions (e.g. Ofman & Thompson 2002) There are many cases of fast mode waves that have been observed in the low corona in association with eruptions (Veronig et al. 2011; Liu et al. 2018), but there has been less work on this phenomena at quiet times. Another possibility is that waves generated by the field reconfiguration propagate through the active region with a temperature-dependent dispersion, for example via fast mode waves. For the fast mode (or any other temperature-dependent mode), the observed frequencies would differ in the AIA filters. There was no clear temperature dependence in the periodicities for our event, suggesting that this mechanism was not operating. Magnetic field reconnection may also occur in an inherently periodic fashion (Van Doorsselaere et al. 2016; Nakariakov & Melnikov 2009). In this case, the AIA emission represents the heating and cooling associated with these periodic reconnection events, and all the AIA filters, as well as the radio data, should exhibit the same frequency, as was the case in our event.

It is possible for the photosphere to be the ultimate source of the periodicity in either case. De Moortel et al. (2002) found different oscillation periods for coronal loops with footpoints inside sunspots than for ones with footpoints outside sunspots. They concluded that the waves were not associated with flares, but rather with a driver whose effects propagated up through the transition region. Correlations observed by Li et al. (2015) between soft X-rays and EUV jets at ~5 min periods led them to suggest that photospheric *p*-modes may lead to periodic reconnection in the corona. De Pontieu et al. (2005) modeled magnetic flux tubes and showed that 5 min *p*-mode waves propagated into the corona, and they could thus be the source of 5 min periodicities in coronal wavelengths. The possibility that the photosphere is the source of the periodicity that we observe is consistent with the correlation of the Type III burts and the EUV with the HMI brightness.

A definitive determination of whether the periodicities in the EUV observed in our event were due to MHD waves, periodic jetting or another process would require analysis of more complex properties in the AIA data, such as Doppler velocity (De Pontieu & McIntosh 2010; Kupriyanova et al. 2020; De Moortel & Nakariakov 2012). Liu & Ofman (2014) review the identification of wave modes in the AIA data, including fast mode waves, Kelvin-Helmholtz waves, and “mini EUV-waves”. The latter may be relevant for our observations as they represent small-scale, weaker waves that occur more often than larger waves.

Some studies of periodicities of minutes have concluded that a modulation of reconnection due to current sheet oscillations is most consistent with their observations (Kupriyanova et al. 2016), and that the height over which modulated processes occur is inconsistent with MHD waves. Other mechanisms for producing modulated reconnection have been investigated by many researchers. Nakariakov et al. (2006) discuss enhanced reconnection associated with kinetic instabilities driven by currents associated with fast mode waves and the interaction of magnetic loops. Liu et al. (2011) found 3 min fast mode waves correlated with the flare QPP, implying a causal link via wave modulation of reconnection. In a study of ~10–20 s QP in flares and radio waves, Fleishman et al. (2008) compared properties to two models, effects of MHD waves on radio emissions and QP injections of electrons, and concluded that the latter better fit their observations.

Our observations are qualitatively similar to those reported by Innes et al. (2011), although those observations centered on very clearly distinguishable, prominent jets. In the event described herein, periodic behavior is observed most clearly in the 211 Å passband (the same as in the Innes study) but jet behavior is less prominent. There are certainly many jets occurring in the active region, but the EUV oscillations are not limited to one jet-producing region. Similar periodicities occur throughout the active region. Li et al. (2015) describe recurrent jets associated with magnetic flux cancelation and periodic, 5 and 13 min, brightenings in the EUV at the jet base. They conclude that their observations were consistent with modulated reconnection. McIntosh & De Pontieu (2009) provided evidence for 3–5 min periodicities in weak upflows in the transition region in the quiet Sun, which may be linked to similar coronal periodicities.

A very different explanation sometimes put forth for quasi-periodic behavior in flares and Type III radio “storms” is based on avalanche or chaos models (Eastwood et al. 2009; Isliker et al. 1998). In a study of two other intervals in this PSP encounter, Pulupa et al. (2020) found that the power law index of intensity distribution was consistent with that of Eastwood et al. (2009). They found that the waiting time distribution dependence on frequency differed in the two intervals, discussed possible reasons for this, and provided comparisons to other studies. Type III storms with very low amplitude and frequent bursts (~800 h^−1^) have been reported by Tun Beltran et al. (2015) using high time resolution ground-based instruments. They propose that continuous reconnection in the corona in concert with density and temperature inhomogeneities may explain their observations. Studies have predicted a range of periodicities for nanoflares (Viall & Klimchuk 2011; Bradshaw et al. 2012; Klimchuk 2015) depending on parameters, such cooling rates. Utilizing MHD simulations of the time intervals between nanoflare reconnection events, Knizhnik & Reep (2020) found a power law distribution, similar to the conclusion of Eastwood et al. (2009) for Type III storms. The time intervals we observed between acceleration events are very consistent over intervals of many days, and thus not explainable by a process resulting in a power law distribution.

We have reported the first observations of periodic Type III radio bursts by PSP and their correlation with periodic rapid heating and cooling in an active region in several EUV channels of the SDO/AIA, and with variations in sunspot brightness seen in the SDO/HMI. The periods were ~5 min in all wavelengths, and comparable to solar *p*-modes. Similar Type III bursts were also observed by WIND. NuSTAR hard X-rays occurred in association with at least one small microflare in the active region, but they were not directly correlated with the Type III bursts. Because Type III radio bursts are generated by nonthermal electrons, this event provides strong evidence for quasi-periodic small-scale acceleration processes in the corona during quiet times. The periodic Type III bursts were observed for days, suggesting that these periodic electron acceleration events may be important for understanding coronal heating.
